# Parental preferences for treatment: Preliminary report from a randomised comparison of treatment strategies in (early) juvenile idiopathic arthritis (BeSt for Kids trial)

**DOI:** 10.1186/1546-0096-9-S1-P184

**Published:** 2011-09-14

**Authors:** PCE Hissink Muller, I Brederije, DMC Brinkman DMC, CF Allaart, LWA van Suijlekom-Smit, MAJ van Rossum, R ten Cate

**Affiliations:** 1 Department of Pediatric Rheumatology, LUMC, Leiden, The Netherlands; 2 Department of Pediatrics/Pediatric Rheumatology, Erasmus MC, Sophia Children’s Hospital, Rotterdam, The Netherlands; 3 Department of Pediatric Rheumatology, Emma Children’s Hospital, AMC, Amsterdam, The Netherlands

## Aim

To prospectively determine treatment preferences among parents of patients with recent onset juvenile idiopathic arthritis participating in a randomized controlled trial comparing three therapeutic strategies.

## Methods

A questionnaire is taken at the start in all parents of the participants of the BeSt for Kids trial, treated with either initial sequential monotherapy (group 1) with either methotrexate or sulfasalazine, initial combination therapy with methotrexate and tapered prednisone (group 2), or initial combination therapy with methotrexate and etanercept (group 3). ACRp50 is the primary goal, treatment adjustments are made every 3 months to aim at clinical remission on medication from 6 months and onwards. The questionnaire explores parental preferences or dislikes for the initial therapy.

## Results

In total, parents of 31 out of 32 so far included patients (97%) completed the questionnaire. 38% of the parents expressed no preference and 53% of the parents expressed no aversion for a particular treatment group. 41% had hoped for assignment to group 3 and 6% had hoped against assignment to group 3. Primary aversion was highest in the second group with 25% due to disliking having to take prednisone.

## Conclusions

This is the first prospective evaluation of parents’ preferences in newly diagnosed juvenile idiopathic arthritis patients participating in the BeSt for Kids trial. Within the limitations of the small amounts, patients clearly preferred initial combination therapy with etanercept and disliked taking prednisone. After actual exposure and follow up, this questionnaire will be repeated to see if preferences remain the same.

